# Gender differences in lung cancer risk by smoking: a multicentre case–control study in Germany and Italy

**DOI:** 10.1054/bjoc.1999.0904

**Published:** 1999-12-08

**Authors:** M Kreuzer, P Boffetta, E Whitley, W Ahrens, V Gaborieau, J Heinrich, K H Jöckel, L Kreienbrock, S Mallone, F Merletti, F Roesch, P Zambon, L Simonato

**Affiliations:** GSF – Institute of Epidemiology, Ingolstaedter Landstr. 1, Neuherberg, 85764, Germany; BfS – Institute of Radiation Hygiene, Ingoldstaedter Landstr. 1, Neuherberg, 85764, Germany; IARC, International Agency for Research on Cancer, 150 Cours Albert-Thomas, Lyon Cedex 08, 69372, France; Department of Social Medicine, University of Bristol, Canynge Hall, Whiteladies Road, Bristol, BS8 2PR, UK; Bremen Institute for Prevention and Social Medicine, Gruenenstr. 120, Bremen, 28199, Germany; University Clinics of Essen, Institute for Medical Informatics, Biometry and Epidemiology and West German Cancer Center Essen (WTZE), Hufelandstr. 55, Essen, 45122, Germany; Epidemiology Unit Latium Region, Via di S Constanza 53, Rome, 0198 Italy; Unit of Cancer Epidemiology, University of Turin, Via Santena 7, Turin, 10126, Italy; Venetian Cancer Registry, Via Gattamelata 64, Padua, 35128, Italy

**Keywords:** lung cancer, case–control study, smoking, histology

## Abstract

Several studies in the past have shown appreciably higher lung cancer risk estimates associated with smoking exposure among men than among women, while more recent studies in the USA report just the opposite. To evaluate this topic in a European population we conducted a case–control study of lung cancer in three German and three Italian centres. Personal interviews and standardized questionnaires were used to obtain detailed life-long smoking and occupational histories from 3723 male and 900 female cases and 4075 male and 1094 female controls. Lung cancer risk comparing ever-smokers with never-smokers was higher among men (odds ratios (OR) adjusted for age and centre = 16.1, 95% confidence interval (CI) 12.8–20.3) than among women (OR = 4.2, CI 3.5–5.1). Because the smoking habits of women were different from men, we conducted more detailed analyses using comparable levels of smoking exposure. After restriction to smokers and adjustment for other smoking variables, risk estimates did not differ appreciably between genders. The analysis of duration of smoking (0–19, 20–39, 40+ years) adjusted for cigarette consumption and time since quitting smoking revealed similar risk estimates in men (OR = 1.0, 3.3 [CI 2.6–4.2], 4.1 [CI 3.1–5.6]) and women (OR = 1.0, 2.7 [CI 1.7–4.1], 3.3 [CI 1.9–5.8]). The same was true of the analysis of average or cumulative smoking consumption, and also of analyses stratified by different histological types. We conclude that for comparable exposure to tobacco smoke, the risk of lung cancer is comparable in women and men. © 2000 Cancer Research Campaign


*British Journal of Cancer* (2000) **82**(1), 227–233
© 2000 Cancer Research Campaign
Article no. bjoc.1999.0904

### Gender differences in lung cancer risk by smoking: a multicentre case–control study in Germany and Italy

**M Kreuzer[1,2], P Boffetta[3], E Whitley[4], W Ahrens[5,6], V Gaborieau[3], J Heinrich[1], KH Jöckel[6], L Kreienbrock[1], S Mallone[7], F Merletti[8], F Roesch[1,3], P Zambon[9] and L Simonato[9]**

[1]GSF – Institute of Epidemiology, Ingolstaedter Landstr. 1, 85764 Neuherberg, Germany; [2]BfS – Institute of Radiation Hygiene, Ingoldstaedter Landstr. 1, 85764 Neuherberg, Germany; [3]IARC, International Agency for Research on Cancer, 150, Cours Albert-Thomas, 69372 Lyon Cedex 08, France; [4]University of Bristol, Department of Social Medicine, Canynge Hall, Whiteladies Road, Bristol BS8 2PR, UK; [5]Bremen Institute for Prevention and Social Medicine, Gruenenstr. 120, 28199 Bremen, Germany; [6]University Clinics of Essen, Institute for Medical Informatics, Biometry and Epidemiology, and West German Cancer Center Essen (WTZE), Hufelandstr. 55, 45122 Essen, Germany; [7]Epidemiology Unit Latium Region, Via di S Constanza, 53, 0198 Rome, Italy; [8]Unit of Cancer Epidemiology, University of Turin, Via Santena, 7, 10126 Turin, Italy; [9] Venetian Cancer Registry, Via Gattamelata, 64, 35128 Padua, Italy

**Summary** Several studies in the past have shown appreciably higher lung cancer risk estimates associated with smoking exposure among men than among women, while more recent studies in the USA report just the opposite. To evaluate this topic in a European population we conducted a case–control study of lung cancer in three German and three Italian centres. Personal interviews and standardized questionnaires were used to obtain detailed life-long smoking and occupational histories from 3723 male and 900 female cases and 4075 male and 1094 female controls. Lung cancer risk comparing ever-smokers with never-smokers was higher among men (odds ratios (OR) adjusted for age and centre = 16.1, 95% confidence interval (CI) 12.8–20.3) than among women (OR = 4.2, CI 3.5–5.1). Because the smoking habits of women were different from men, we conducted more detailed analyses using comparable levels of smoking exposure. After restriction to smokers and adjustment for other smoking variables, risk estimates did not differ appreciably between genders. The analysis of duration of smoking (0–19, 20–39, 40+ years) adjusted for cigarette consumption and time since quitting smoking revealed similar risk estimates in men (OR = 1.0, 3.3 [CI 2.6–4.2], 4.1 [CI 3.1–5.6]) and women (OR = 1.0, 2.7 [CI 1.7–4.1], 3.3 [CI 1.9–5.8]). The same was true of the analysis of average or cumulative smoking consumption, and also of analyses stratified by different histological types. We conclude that for comparable exposure to tobacco smoke, the risk of lung cancer is comparable in women and men. © 2000 Cancer Research Campaign

**Keywords:** lung cancer; case–control study; smoking; histology

Lung cancer is the leading cancer among men in Europe, and the fourth most common among women (Parkin et al, 1997). During the last few decades, lung cancer incidence and mortality among men in Western European countries has stabilized or is declining. This is in contrast to Eastern European countries where rates show consistent and continued increases (Coleman et al, 1993). Lung cancer mortality among women in most European countries is continuing to rise at a steady rate (Coleman et al, 1993; Evstifeeva et al, 1997). Cigarette smoking is the major cause of lung cancer in both genders. In European countries in the past, fewer women than men were smokers, and women who did smoke usually started at a later age, smoked fewer cigarettes per day and inhaled less deeply than men (Nicolaides-Bouman et al, 1993). In more recent years the smoking pattern of women in Western Europe has converged towards the pattern in males, and this is particularly apparent in young subjects. If current trends continue, lung cancer rates among women may be expected to approach those of men. Franceschi et al (1994) report that lung cancer mortality rates in young (20–44 years) men and women were already nearly equal in Scandinavian countries during the late 1980s. The same pattern is seen in 1988–1992 incidence rates in Norway, Sweden and Denmark (Parkin et al, 1997).

*Received 18 January 1999*
*Revised 1 April 1999*
*Accepted 1 April 1999*

*Correspondence to:* M Kreuzer, Federal Office of Radiation Protection, Institute of Radiation Hygiene, Ingolstaedter Landstr. 1, D-85764 Neuherberg, Germany

The majority of studies have reported considerably higher risks of lung cancer in male smokers compared with female smokers (Haenszel and Taeuber, 1964; Hammond, 1966; Doll et al, 1980; Doll and Peto, 1981). This result may reflect a different susceptibility to the carcinogenic effect of tobacco, or it may also be a result of the lower exposure to tobacco smoke among smoking women. In contrast, several more recent studies have indicated that the relative risk of lung cancer associated with smoking may be even higher in female smokers than in male smokers (Lubin and Blot, 1984; McDuffie et al, 1987; Brownson et al, 1992; Zang and Wynder, 1992, 1996; Harris et al 1993; Osann et al, 1993; Risch et al, 1993). These studies were mainly conducted in the US, where the exposure of women to tobacco carcinogens is already approaching, and may soon surpass, that of men (Ries et al, 1994; Baldini and Strauss, 1997).

The aim of this study was to evaluate, in a European population, whether women and men have a different susceptibility to tobacco carcinogens. The multicentre case–control study provides a large number of female and male cases and controls and a detailed quantification of life-long smoking exposure for each participant.

**Table 1** Characteristics of study population by case–control status and gender

| | Men | | | | Women | | | |
|---|---|---|---|---|---|---|---|---|
| | Cases | | Controls | | Cases | | Controls | |
| | *n* | % | *n* | % | *n* | % | *n* | % |
| Age | | | | | | | | |
| < 40 | 80 | 2.2 | 77 | 1.9 | 19 | 2.1 | 31 | 2.8 |
| 40–49 | 340 | 9.1 | 327 | 8.0 | 126 | 14.0 | 124 | 11.3 |
| 50–59 | 1215 | 32.6 | 1262 | 31.0 | 260 | 28.9 | 322 | 29.4 |
| 60–69 | 1600 | 43.0 | 1821 | 44.7 | 369 | 41.0 | 409 | 37.4 |
| 70 + | 488 | 13.1 | 588 | 14.4 | 126 | 14.0 | 208 | 19.0 |
| Centre | | | | | | | | |
| Germany I | 542 | 14.6 | 547 | 13.4 | 159 | 17.7 | 154 | 14.1 |
| Germany II | 1438 | 38.6 | 1426 | 35.0 | 402 | 44.7 | 417 | 38.1 |
| Germany III | 611 | 16.4 | 753 | 18.5 | 116 | 12.8 | 127 | 11.6 |
| Italy I | 408 | 11.0 | 573 | 14.1 | 89 | 9.9 | 175 | 16.0 |
| Italy II | 447 | 12.0 | 527 | 12.9 | 82 | 9.1 | 123 | 11.2 |
| Italy III | 277 | 7.4 | 249 | 6.1 | 52 | 5.8 | 98 | 8.9 |
| Education[a] | | | | | | | | |
| Mean | 7.87 | | 8.58 | | 8.00 | | 8.24 | |
| Standard deviation | 2.16 | | 2.61 | | 2.13 | | 2.64 | |
| Histological type | | | | | | | | |
| Small-cell carcinoma | 724 | 19.4 | – | – | 188 | 20.9 | – | – |
| Adenocarcinoma | 644 | 17.3 | – | – | 304 | 33.8 | – | – |
| Squamous cell cancer | 1859 | 49.9 | – | – | 253 | 28.1 | – | – |
| Other[b] | 496 | 13.3 | – | – | 153 | 17.0 | – | – |
| Occupational exposure to known or suspected lung carcinogens | | | | | | | | |
| Never exposed | 2068 | 55.5 | 2753 | 67.6 | 763 | 84.8 | 1005 | 91.9 |
| Ever exposed | 1655 | 44.4 | 1322 | 32.4 | 137 | 15.2 | 89 | 8.1 |
| **Totals** | 3723 | 100 | 4075 | 100 | 900 | 100 | 1094 | 100 |

[a]Years of school attendance; [b]large cell carcinoma or mixed types.

### MATERIALS AND METHODS

We used the data of six case–control studies of lung cancer among men and women. These data were part of a pooled analysis of European case–control studies of lung cancer (Simonato et al, 1997). Two study centres were from West Germany (‘Germany I’ (Jöckel et al, 1998): cities of Bremen and Frankfurt and surroundings; ‘Germany II’: Eastern Bavaria, Saarland, Eifel, Northrhine-Westfalia), one from former GDR (‘Germany III’: Thuringia and Saxony) and three from Italy (‘Italy I’: Turin; ‘Italy II’: surroundings of Venice = Venetian Region, North East of Italy, ‘Italy III’: Rome). Cases and controls were enrolled between 1988 and 1994. Controls were community-based with the exception of the Italy III centre, which used hospital controls. Community controls were obtained by random selection of population registries or random digit dialling. Hospital controls did not include patients admitted for smoking-related diseases. The upper age limit for inclusion was 74 years in cases and controls. Only histologically or cytologically confirmed lung cancer patients were included as cases.

A common questionnaire was used to determine demographic variables, life-long smoking history and exposure to known or suspected occupational carcinogens. Subjects were defined as smokers if they had ever smoked at least one cigarette per day for at least 1 year. Detailed information was collected on type of tobacco, average daily consumption, duration and filter usage for each period of smoking (defined by a change in either quantity or type of tobacco). Smokers who ever smoked products other than cigarettes (e.g. cigars, pipes or cigarillos) were excluded from this analysis because of the small numbers among women. Current smokers were defined as those who were still smoking 2 years before interview. Smokers who had stopped 2 or more years before interview were defined as ex-smokers.

Subjects were classed as having been occupationally exposed to known or suspected lung carcinogens if they had worked for at least 6 months in a job entailing exposure to recognized or suspected lung carcinogens, mostly based on the IARC Monographs Programme (Simonato and Saracci, 1983; Boffetta et al, 1995; Ahrens and Merletti, 1998).

Cases and controls were frequency matched on gender, age and area of residence in five centres, and individually in one (Germany I). As conditional and unconditional logistic regression models (Breslow and Day, 1980) gave nearly identical estimates, odds ratios (ORs) and 95% confidence intervals (CIs) from an unconditional model, using the SAS procedure GENMOD (SAS Institute, 1989), are presented here. All analyses were adjusted for age and centre. Additional potential confounders were occupation (never vs ever exposed to a known or suspected carcinogen) and educational level (in three categories). There was no clear evidence of confounding and results are presented adjusted for age and centre only. Likelihood ratio tests of the interaction between gender and smoking exposure were performed using a model with data from both genders combined and adding an interaction term. Tests for linear trends were performed by using continuous data excluding never smokers.

© 2000 Cancer Research Campaign

**Table 2** Smoking habits of study population by case–control status and gender

| | Men | | Women | |
|---|---|---|---|---|
| | Cases | Controls | Cases | Controls |
| % never smokers | | | | |
| < 50 years of age | 5.0 | 33.9 | 11.7 | 49.6 |
| 50+ years of age | 1.8 | 24.6 | 35.6 | 67.9 |
| Total | 2.1 | 25.6 | 31.8 | 65.4 |
| Total number of ever smokers | 3642 | 3032 | 614 | 379 |
| Duration of smoking (years) | | | | |
| Mean | 38.5 | 30.5 | 33.2 | 26.1 |
| Standard deviation | 10.0 | 14.0 | 11.6 | 14.3 |
| Average daily consumption (cig/day) | | | | |
| Mean | 20.8 | 16.6 | 15.9 | 11.1 |
| Standard deviation | 9.9 | 10.1 | 9.0 | 7.8 |
| Cumulative consumption (packyears) | | | | |
| Mean | 40.2 | 26.6 | 27.1 | 15.9 |
| Standard deviation | 22.0 | 20.8 | 17.7 | 15.5 |
| Age started smoking (years) | | | | |
| Mean | 17.8 | 18.7 | 22.2 | 23.9 |
| Standard deviation | 4.0 | 5.1 | 7.9 | 8.8 |
| % of smokers who used a filter | | | | |
| Filter cigarettes only | 18.2 | 23.3 | 57.0 | 67.3 |
| Non filter cigarettes (pure or mixed with filter) | 79.5 | 74.9 | 39.3 | 28.5 |
| Missing | 2.3 | 1.8 | 3.8 | 4.2 |

cig/day: cigarettes per day.

### RESULTS

A total of 4623 lung cancer cases (3723 males and 900 females) and 5169 controls (4075 males and 1094 females) were included in the analysis. Table 1 shows the basic characteristics for both genders. The relative frequency of squamous cell carcinoma was substantially higher in men (50%) than in women (28%), while adenocarcinoma was more common among women (34%) than among men (17%) and small cell carcinoma had the same frequency. Controls tended to be more highly educated than cases in both genders. More cases than controls were occupationally exposed to carcinogens among both genders, with much higher proportions among men (44% of male cases were occupationally exposed to known or suspected lung carcinogens compared with 15% of female cases).

The smoking habits of the study population are shown in Table 2. Women generally had less exposure to cigarettes than men among both lung cancer cases and controls. The proportion of female controls who had never smoked was 65% as compared with 26% of male controls. These proportions decreased markedly in women aged less than 50 (50% of controls) and slightly increased in young men (34% of controls). On average, regardless of case–control status, women started smoking around 5 years later than men, smoked around five cigarettes fewer per day, and smoked for around 5 years less. About 70% of female smoking controls used only filter cigarettes as compared to 23% of males.

Table 3 gives the gender-specific ORs for lung cancer comparing subjects who ever smoked cigarettes with those who never smoked cigarettes. Separate ORs are given for different histological types, for current and ex-smokers, and for subjects younger than 50. Overall, male smokers showed a 16-fold increase in lung cancer risk compared to an OR of 4.2 in female smokers. There was considerable variation in the risks for the different histological types but, as expected, the risk for adenocarcinoma was lower than that for squamous cell and small-cell carcinoma in both genders. ORs were systematically higher for men than for women, but the male to female ratio was highest for squamous cell carcinoma (5.6), followed by small-cell carcinoma (4.2) and adenocarcinoma (2.3). Restriction to current smokers gave higher ORs than for all smokers combined in both genders and the male to female ratio remained unchanged. The restriction to subjects aged less than 50 gave a lower OR in men and a higher OR in women, resulting in a much smaller male to female ratio (1.4). A test of interaction between smoking and gender was not significant in the younger age group.

Table 4 summarizes the risk of lung cancer according to duration of smoking and cigarette consumption. Overall, the ORs for men were two- to sixfold higher than those for women at each exposure level. In contrast, restriction of the analyses to young subjects (< 50 years) showed very similar risk estimates by gender.

Since the lower proportion of never-smokers among male lung cancer cases may inflate the ORs among men, we repeated the analyses with never-smokers excluded and using the smallest smoking exposure as the reference category (Table 5). After reciprocal adjustment of smoking variables, no systematic differences in risk between men and women were observed. The trend for duration of smoking was slightly stronger in men than in women, while the opposite effect was seen for average consumption. There

**Table 3** Lung cancer risk for ever having regularly smoked cigarettes (reference category never-smokers)

| | Men | | | | Women | | | | |
|---|---|---|---|---|---|---|---|---|---|
| | No. of never/ever smokers | | OR[a] | 95% CI[b] | No. of never/ever smokers | | OR[a] | 95% CI[b] | |
| | Cases | Controls | | | Cases | Controls | | | *P* for interaction[c] |
| All histological types | 81/3642 | 1043/3032 | 16.1 | 12.8–20.3 | 286/614 | 715/379 | 4.2 | 3.5–5.1 | < 0.0001 |
| Squamous carcinoma | 15/1844 | 1043/3032 | 42.3 | 25.3–70.7 | 54/199 | 715/379 | 7.5 | 5.4–10.5 | < 0.0001 |
| Small-cell carcinoma | 7/717 | 1043/3032 | 40.1 | 19.0–84.8 | 32/156 | 715/379 | 9.5 | 6.2–14.5 | < 0.0001 |
| Adenocarcinoma | 41/603 | 1043/3032 | 5.1 | 3.7–7.1 | 143/161 | 715/379 | 2.2 | 1.6–2.8 | < 0.0001 |
| Other cell type | 18/478 | 1043/3032 | 9.5 | 5.9–15.3 | 55/98 | 715/379 | 3.3 | 2.3–4.8 | < 0.0001 |
| Current smokers | 81/2525 | 1043/1219 | 28.1 | 22.2–35.7 | 286/501 | 715/207 | 6.5 | 5.2–8.1 | < 0.0001 |
| Ex-smokers | 81/1117 | 1043/1813 | 7.5 | 5.9–9.6 | 286/113 | 715/172 | 1.7 | 1.3–2.3 | < 0.003 |
| Subjects below 50 | 21/399 | 137/267 | 10.0 | 6.1–16.2 | 17/128 | 77/78 | 7.3 | 3.9–13.5 | = 0.4 |

[a]OR: odds ratio adjusted for age and centre; [b]95% CI: 95% confidence interval; [c]*P*-value for likelihood ratio test of the interaction between gender and smoking exposure.

*© 2000 Cancer Research Campaign*

**Table 4** Lung cancer risk by different smoking variables (reference category never smokers)

| | | Men | | | | Women | | | | *P* for interaction[b] |
|---|---|---|---|---|---|---|---|---|---|---|
| | | Cases | Controls | OR[a] | 95% CI | Cases | Controls | OR[a] | 95% CI | |
| All subjects | Duration of smoking (years) | | | | | | | | | |
| | < 20 | 143 | 720 | 2.4 | 1.8–3.3 | 71 | 139 | 1.2 | 0.9–1.7 | |
| | 20–39 | 1720 | 1398 | 16.4 | 12.9–20.9 | 347 | 165 | 5.3 | 4.2–6.8 | |
| | 40+ | 1779 | 914 | 39.1 | 30.4–50.3 | 196 | 75 | 7.0 | 5.1–9.5 | < 0.0001 |
| | *P* for linear trend | | | < 0.0001 | | | | < 0.0001 | | |
| | Average daily consumption | | | | | | | | | |
| | < 15 cig/day | 906 | 1412 | 8.6 | 6.7–10.9 | 311 | 278 | 2.9 | 2.4–3.6 | |
| | 15–29 cig/day | 2197 | 1341 | 21.7 | 17.1–27.4 | 257 | 91 | 7.8 | 5.8–10.3 | |
| | 30 + cig/day | 539 | 279 | 25.4 | 19.4–33.3 | 46 | 10 | 13.8 | 6.8–28.1 | < 0.0001 |
| | *P* for linear trend | | | < 0.0001 | | | | < 0.0001 | | |
| Subjects | Duration of smoking (years) | | | | | | | | | |
| < 50 years | < 20 | 41 | 113 | 2.1 | 1.2–3.8 | 23 | 45 | 2.2 | 1.0–4.7 | |
| | 20–39 | 358 | 154 | 16.4 | 9.9–27.2 | 105 | 33 | 14.4 | 7.2–28.6 | |
| | *P* for linear trend | | | < 0.0001 | | | | < 0.0001 | | = 1.0 |
| | Average daily consumption | | | | | | | | | |
| | < 15 cig/day | 59 | 91 | 4.3 | 2.4–7.5 | 45 | 53 | 3.8 | 1.9–7.6 | |
| | 15–29 cig/day | 262 | 150 | 11.6 | 7.0–19.3 | 68 | 22 | 15.0 | 7.0–32.0 | |
| | 30 + cig/day | 78 | 26 | 20.7 | 10.8–39.6 | 15 | 3 | 23.4 | 5.9–92.3 | = 0.8 |
| | *P* for linear trend | | | < 0.0001 | | | | < 0.0001 | | |

cig/day = cigarettes per day; [a]OR adjusted for age and centre; [b]likelihood ratio test of the interaction between gender and smoking exposure

**Table 5** Lung cancer risk by different smoking variables (analysis restricted to ever smokers)

| | Men | | Women | | *P* for interaction[c] |
|---|---|---|---|---|---|
| | OR | 95% CI | OR | 95% CI | |
| Duration of smoking[a] | | | | | |
| < 20 years | 1.0 | | 1.0 | | |
| 20–39 years | 3.2 | 2.5–4.0 | 2.7 | 1.7–4.1 | |
| 40 + years | 4.1 | 3.1–5.6 | 3.3 | 1.9–5.8 | = 0.1 |
| *P* for linear trend | < 0.0001 | | < 0.0001 | | |
| Average daily consumption[b] | | | | | |
| < 15 cig/day | 1.0 | | 1.0 | | |
| 15–29 cig/day | 2.0 | 1.8–2.2 | 2.0 | 1.4–2.7 | |
| 30+ cig/day | 2.4 | 2.0–2.8 | 3.4 | 1.6–7.3 | = 0.6 |
| *P* for linear trend | < 0.0001 | | < 0.0001 | | |
| Cumulative consumption (pack-years)[c] | | | | | |
| < 20 | 1.0 | | 1.0 | | |
| 20–39 | 2.6 | 2.3–3.0 | 2.8 | 2.0–3.9 | |
| 40 + | 3.9 | 3.3–4.6 | 4.0 | 2.5–6.5 | = 0.9 |
| *P* for linear trend | < 0.0001 | | < 0.0001 | | |
| Time since stopping smoking[d] | | | | | |
| Current | 1.0 | | 1.0 | | |
| 2–9 years | 0.7 | 0.6–0.8 | 0.5 | 0.3–0.7 | |
| 10–19 years | 0.2 | 0.2–0.3 | 0.2 | 0.1–0.3 | |
| 20+ years | 0.1 | 0.1–0.1 | 0.2 | 0.1–0.3 | = 0.044 |
| *P* for linear trend | < 0.0001 | | < 0.0001 | | |

cig/day = cigarettes per day. [a]OR adjusted for age, centre, average amount and time since stopping smoking; [b]OR adjusted for age, centre, duration and time since stopping smoking; [c]OR adjusted for age, centre, time since stopping smoking; [d]OR adjusted for age, centre and average amount; [e]Likelihood ratio test of the interaction between gender and smoking exposure adjusted for age, centre, duration/average amount and time since stopping smoking.

were no gender differences in lung cancer risk by cumulative consumption (pack-years) or time since stopping smoking.

These analyses were also carried out for each of the different histological types (Table 6). The OR for each cell subtype according to duration of smoking or cigarette consumption was similar in men and women and no substantial differences in lung cancer risk by smoking associated with different cell types were observed.

### DISCUSSION

The aim of this detailed analysis of the data from a European multicentre study was to further explore gender differences in lung cancer risk associated with smoking. The main strength of this study is its size with 3723 male lung cancer cases, 900 female cases and a correspondingly large number of controls. This large sample size allowed us to conduct analyses on subgroups such as

*© 2000 Cancer Research Campaign*

**Table 6** Lung cancer risk by various smoking variables (reference category weak smokers) and histological type

| | Men | | | Women | | |
|---|---|---|---|---|---|---|
| **No. of cases/controls** | **SCLC 717/3032 OR[a]** | **SqCC 1844/3032 OR[a]** | **AC 603/3032 OR[a]** | **SCLC 156/379 OR[a]** | **SqCC 199/379 OR[a]** | **AC 161/379 OR[a]** |
| Duration of smoking (years) | | | | | | |
| < 20 | 1.0 | 1.0 | 1.0 | 1.0 | 1.0 | 1.0 |
| 20–39 | 3.3 | 3.2 | 3.4 | 3.9 | 2.4 | 2.2 |
| 40 + | 3.8 | 4.1 | 4.0 | 3.6 | 4.2 | 2.3 |
| Average daily consumption (cig/day) | | | | | | |
| < 15 | 1.0 | 1.0 | 1.0 | 1.0 | 1.0 | 1.0 |
| 15–29 | 2.2 | 2.2 | 1.6 | 2.0 | 1.8 | 2.1 |
| 30+ | 2.6 | 3.0 | 1.6 | 4.7 | 3.8 | 4.6 |

SCLC: small-cell lung cancer; SqCC: squamous cell carcinoma; AC: adenocarcinoma; cig/day: cigarettes per day. [a]OR: odds ratio adjusted for age, centre and duration/average amount and time since quitting smoking.

young subjects and specific histological types. An additional strong methodological aspect is the detailed ascertainment of life-time smoking exposure by standardized questionnaire. Additional information was also available on potential confounders such as occupational exposures and educational levels.

Results of the present study indicate that relative risk estimates of lung cancer due to smoking did not differ substantially between men and women. In our first approach (never-smokers as reference category and no additional adjustment for other smoking variables), we obtained fourfold higher risk estimates for various indices of smoking in men compared to women, independently of histological type. However, because men were more likely to be smokers, smoked more cigarettes, started to smoke earlier, smoked for longer and used fewer filter cigarettes; a similar or even higher susceptibility of women was likely to have been masked by their lower exposures. The same analyses restricted to young subjects showed little or no difference in lung cancer risk between men and women. This is likely to reflect the fact that young birth cohorts show less gender differences in smoking patterns than older birth cohorts (Kreuzer et al, 1998). The reduction in the difference in OR due to cigarette smoking between men and women in young as compared to older age groups strongly argues against the hypothesis of a lower susceptibility of women to lung cancer.

In addition, our second approach (reciprocal adjustment for smoking variables and exclusion of never-smokers) removed the differences in risk between men and women. ORs associated with comparable exposure levels of duration of smoking and cumulative consumption were similar in the two genders, regardless of histological type. ORs for duration of smoking did not change substantially after further adjustment for average consumption, but strongly decreased after additional adjustment for time since quitting. For example, in the highest category of duration of smoking risk estimates were about two times higher in men compared to women (OR = 16.3 among men, OR = 7.4 among women), this remained after adjustment for average consumption (15.2 vs 6.7) and decreased to a nearly equal risk ratio between men and women after considering the confounder time since stopping smoking (4.1 vs 3.3, Table 5).

Several studies addressing the association of smoking and lung cancer with respect to gender differences have provided conflicting results. Recent studies in the US in the 1990s reported a consistently higher susceptibility to lung carcinogens in women than in men (McDuffie et al, 1987; Brownson et al, 1992; Zang and Wynder, 1992, 1996; Harris et al, 1993; Osann et al, 1993; Risch et al, 1993). Results of a case–control study conducted in Canada between 1980 and 1985 suggested that female smokers were about three times more likely than men to develop lung cancer (Risch et al, 1993). Another, larger, case–control study with more than 4000 lung cancer cases reported that female smokers were 1.5–2 times more likely to develop cancer than men (Harris et al, 1993). A higher RR of lung cancer for female smokers was reported by Zang and Wynder for every level of tar exposure (1992). A re-analysis of this data (Zang and Wynder, 1996) with more detailed exposure quantification and more defined histological types again showed that ORs for cumulative exposure to cigarette smoking were 1.2- to 1.7-fold higher in women than in men for the three major histological types. A large case–control study conducted between 1976 and 1980 in Western Europe with 6920 male and 884 female lung cancer patients reported a steeper increase in risk for women with increasing duration, quantity and intensity of smoking than in men for each histological type (Lubin and Blot, 1984).

The American Cancer Society's Second Cancer Prevention Study (CPS-II), which has been following more than a million Americans since 1982 (United States Public Health Service, 1989), has found that male smokers are twice as likely to die from lung cancer as female smokers (RR = 22.4 in men, RR = 11.9 in women). Several other studies show no clear gender differences according to various dose measures of cigarette smoking (Humble et al, 1985; Schoenberg et al, 1989; Osann et al, 1993). The most recent prospective, population-based study, conducted between 1964 and 1994 in Copenhagen, Denmark and following about 30 000 subjects, showed no differences in lung cancer risk between female and male smokers (Prescott et al, 1998). The ratio between female and male smokers' RRs of developing lung cancer was 0.8 after adjusting for pack-years, age and study population.

These conflicting results have encouraged much discussion (Taubes, 1993; Hoover, 1994; McDuffie, 1994; Wilcox, 1994; Weiss, 1996; Baldini and Strauss, 1997). The criticisms most often levelled at studies showing higher risks for women than for men is that different baseline risks are likely in non-smoking women compared with non-smoking men (e.g. due to fewer work-related exposures to lung carcinogens). To minimize the possible effect of different occupational baseline risks in the two genders in the

*© 2000 Cancer Research Campaign*

present study, we repeated all analyses excluding subjects with occupational exposures to known or suspected lung carcinogens (about 40% of men and 10% of women). There were no material differences in the overall results and there was no evidence of a difference in risk between men and women. For example, the OR in the highest category of average consumption, as in Table 5, was 2.6 (CI 2.2–3.3) in men and 2.8 (CI 1.3–6.2) in women.

The small non-significant differences in excess rate observed between men and women in Table 5 may simply be due to chance. Another possible explanation are different ways of smoking, e.g. men inhaling more deeply than women. However, additional adjustment for depth of inhalation exerted only minor effects. As an example, the OR for the highest category of duration of smoking (OR = 4.1, CI 3.1–5.6) was modified to OR = 3.9 (CI 2.9–5.2) in the group of men and the OR = 3.3 (CI 1.9–5.8) to 2.9 (1.6–5.2) in the group of women respectively. In addition, several studies have found that the exposure to tar and nicotine in men is higher than in women. Since we did not adjust for lifelong tar consumption, the ORs for women compared with those of men may be underestimated. We do not have this information, but it is unlikely to totally explain our results. Additional adjustment for filter or non filter cigarettes at least did not change any results.

Another possible source of error in exposure assessment is selective recall bias. If female controls underreported their smoking exposure with respect to female cases in a proportion substantially greater than that of male controls and cases, this would result in an overestimate of lung cancer risk in women as compared to men. The use of face-to-face interviews by trained interviewers using a standardized questionnaire should have reduced recall bias to a minimum. However, a possible bias due to differential inaccuracies cannot be excluded. It is impossible to obtain direct estimates of the validity of information regarding past smoking habits. To get measures of reproducibility Morabia et al (1990) and Donato et al (1998) conducted re-interviews in a subsample of their study participants. Both found a high reliability for responses related to smoking, and no important gender differences were reported (Donato et al, 1998).

In conclusion, the results of the present study do not support a gender difference in susceptibility to lung cancer from similar levels of tobacco smoking. This holds true for all histological types and does not reflect confounding by differences in occupational exposures between men and women.

### ACKNOWLEDGEMENTS

This study was partially supported by the following grants: European Commission, DG-XII, Biomed-1 programme (contract No. BMH1-CT93-1047) for the coordination; in Germany 1, the Federal Ministry for Education, Science, Research and Technology (grant No. 01 HK 546); in Germany 2 and 3, the Federal Office of Radiation Protection (grant No. St sch 1066, 4047, 4074/1, 4006, 4112), in Italy 1, MURST, the Italian Association for Cancer Research (AIRC), and the Regione Piemonte-Ricerca Finalizzata, in Italy 2, the National Research Council (contract No. 91.00327.CT04) and the Italian Association for Cancer Research; in Italy 3, the Regione Lazio. We thank the following persons for their contribution to the study in the different centres: Germany 1 – H Pohlabeln, I Jahn; Germany 2 – M Gerken, G Dingerkus; Germany 3 – G Wölke, K-M Müller; Italy 1 – S Massacesi, M Tedeschi, M Artom, G Peyrano, R Richiardi; Italy 3 – F Anatra, T Trequattrini.

### REFERENCES

Ahrens W and Merletti F (1998) A standard tool for the analysis of occupational lung cancer in epidemiological studies. *Int J Occup Environ Health* **4**: 236–240

Baldini EH and Strauss GM (1997) Women and lung cancer: waiting to exhale. *Chest* **112**: 229S–234S

Boffetta P, Kogevinas M, Simonato L, Wilbourn J and Saracci R (1995) Current perspectives on occupational cancer risks. *Int J Occup Environ Health* **1**: 315–325

Breslow NE and Day NE (1980) *Statistical Methods in Cancer Research, Vol. 1: The Analysis of Case-Control-Studies*. International Agency for Research on Cancer: Lyon

Brownson RC, Chang JC and Davis JR (1992) Gender and histologic type variations in smoking-related risk of lung cancer. *Epidemiology* **3**: 61–64

Coleman M, Esteve J, Damiecki P, Arslan A and Renard H (1993) *Time Trends in Cancer Incidence and Mortality*. IARC Scientific Publications Vol 121. International Agency for Research on Cancer: Lyon

Doll R and Peto R (1981) The causes of cancer: quantitative estimates of avoidable risks in the United States today. *J Natl Cancer Inst* **66**: 1191–1308

Doll R, Gray R, Hafner B and Peto R (1980) Mortality in relation to smoking: 22 years' observations on female British doctors. *Br Med J* **280**: 967–971

Donato F, Boffetta P, Fazioloi R, Gelatti U and Porru S (1998) Reliability of data on smoking habit and coffee drinking collected by personal interview in a hospital-based case–control study. *Eur J Epidemiol* **14**: 259–267

Evstifeeva TV, Macfarlane GJ and Robertson C (1997) Trends in cancer mortality in central European countries. *Eur J Public Health* **7**: 169–176

Franceschi S, Levi F, Lucchini F, Negri E, Boyle P and La Vecchia C (1994) Trends in cancer mortality in young adults in Europe, 1955–1989. *Eur J Cancer* **30A**: 2096–2118

Haenszel W and Taeuber KE (1964) Lung cancer mortality as related to residence and smoking histories. II. White females. *J Natl Cancer Inst* **32**: 803–838

Hammond EC (1966) Smoking in relation to the death rates of one million men and women. *Natl Cancer Inst Monogr* **19**: 127–204

Harris RE, Zang EA, Anderson JI and Wynder EL (1993) Race and sex differences in lung cancer risk associated with cigarette smoking. *Int J Epidemiol* **22**: 592–599

Hoover DR (1994) Re: Are female smokers at higher risk for lung cancer than male smokers? A case–control analysis by histologic type. *Am J Epidemiol* **140**: 186–187

Humble CG, Samet JM, Pathak DR and Skipper BJ (1985) Cigarette smoking and lung cancer in 'Hispanic' whites and other whites in New Mexico. *Am J Public Health* **75**: 145–148

Jöckel KH, Ahrens W, Jahn I, Pohlabeln H and Bolm-Audorff U (1998) Occupational risk factors for lung cancer: a case–control study in West Germany. *Int J Epidemiol* **27**: 549–560

Kreuzer M, Kreienbrock L, Gerken M, Heinrich J, Brüske-Hohlfeld I, Müller K-M and Wichmann HE (1998) Risk factors for lung cancer in young adults. *Am J Epidemiol* **147**: 1028–1037

Lubin JH and Blot WJ (1984) Assessment of lung cancer risk factors by histologic category. *J Natl Cancer Inst* **73**: 383–389

McDuffie HH (1994) Re: Are female smokers at higher risk for lung cancer than male smokers? A case–control analysis by histologic type. *Am J Epidemiol* **140**: 185–186

McDuffie HH, Klaassen DJ and Dosman JA (1987) Female–male differences in patients with primary lung cancer. *Cancer* **59**: 1825–1830

Morabia A, Moore M and Wynder EL (1990) Reproducibility of food frequency measurements and inferences from a case–control study. *Epidemiology* **1**: 305–310

Nicolaides-Bouman A, Wald N, Forey B and Lee P (1993) *International Smoking Statistics: a Collection of Historical Data from 22 Economically Developed Countries*. Oxford Press: Oxford

Osann KE, Anton-Culver H, Kurosaki T and Taylor T (1993) Sex differences in lung cancer risk associated with cigarette smoking. *Int J Cancer* **54**: 44–48

Parkin DM, Whelan SL, Ferlay J, Raymond L and Young J (1997) *Cancer Incidence in Five Continents*. IARC Scientific Publications Vol 143. International Agency for Research on Cancer: Lyon

Prescott E, Osler M, Hein HO, Borch-Johnson K, Lange P, Schnohr P, Vestbo J and the Copenhagen Center for Prospective Population Studies (1998) Gender and smoking-related risk of lung cancer. *Epidemiology* **9**: 79–83

Ries LA, Miller BA and Hankey BF (1994) *SEER Cancer Statistics Review, 1973–1991: Tables and Graphs*. Bethesda: National Cancer Institute. NIH Publications, 264

© 2000 Cancer Research Campaign

Risch HA, Howe GR, Jain M, Burch JD, Holowaty EJ and Miller AB (1993) Are female smokers at higher risk for lung cancer than male smokers? A case–control analysis by histologic type. *Am J Epidemiol* **138**: 281–293
SAS Institute Inc (1989) *SAS/STAT User's Guide*. SAS Institute: Cary, NC
Schoenberg JB, Wilcox HB, Mason TJ, Bill J and Stemhagen A (1989) Variation in smoking-related lung cancer risk among New Jersey women. *Am J Epidemiol* **130**: 688–695
Simonato L and Saracci R (1983) *Cancer, occupational*. Geneva: ILO: 369–375
Simonato L, Boffetta P, Roesch F, Gaborieau V and Sartorel S (1997) *A Multicentric Case–control Study of the Major Risk Factors for Lung Cancer in Europe with Particular Emphasis on Intercountry Comparison – Final scientific report*. Venetian Tumour Registry: Padua
Taubes G (1993) Claim of higher risk for women smokers attacked. *Science* **262**: 1375
United States Public Health Service (1989) Reducing the health consequences of smoking: 25 years of progress. A report of the Surgeon General. Office on Smoking and Health, DHHS publication no. (CDC) 89-8411
Weiss N (1996) Re: Differences in lung cancer risk between men and women: examination of the evidence. *J Natl Cancer Inst* **88**: 764
Wilcox AJ (1994) Re: Are female smokers at higher risk for lung cancer than male smokers? A case–control analysis by histologic type. *Am J Epidemiol* **140**: 186
Zang EA and Wynder EL (1992) Cumulative tar exposure: a new index for estimating lung cancer risk among cigarette smokers. *Cancer* **70**: 69–76
Zang EA and Wynder EL (1996) Differences in lung cancer risk between men and women: examination of the evidence. *J Natl Cancer Inst* **88**: 183–192

*© 2000 Cancer Research Campaign*
